# Sex differences in dietary intake in British Army recruits undergoing phase one training

**DOI:** 10.1186/s12970-019-0327-2

**Published:** 2019-12-10

**Authors:** Shaun Chapman, Justin Roberts, Lee Smith, Alex Rawcliffe, Rachel Izard

**Affiliations:** 1 HQ Army Recruiting and Initial Training Command, UK Ministry of Defence, Upavon, UK; 2 Cambridge Centre for Sport and Exercise Sciences, School of Psychology and Sport Science, Anglia Ruskin University, East Road, Cambridge, CB1 1PT England

**Keywords:** Dietary intake, Military, Sex differences, Exercise training

## Abstract

**Background:**

British Army Phase One training exposes men and women to challenging distances of 13.5 km·d^− 1^ vs. 11.8 km·d^− 1^ and energy expenditures of ~ 4000 kcal·d^− 1^ and ~ 3000 kcal·d^− 1^, respectively. As such, it is essential that adequate nutrition is provided to support training demands. However, to date, there is a paucity of data on habitual dietary intake of British Army recruits. The aims of this study were to: (i) compare habitual dietary intake in British Army recruits undergoing Phase One training to Military Dietary Reference Values (MDRVs), and (ii) establish if there was a relative sex difference in dietary intake between men and women.

**Method:**

Researcher led weighed food records and food diaries were used to assess dietary intake in twenty-eight women (age 21.4 ± 3.0 yrs., height: 163.7 ± 5.0 cm, body mass 65.0 ± 6.7 kg), and seventeen men (age 20.4 ± 2.3 yrs., height: 178.0 ± 7.9 cm, body mass 74.6 ± 8.1 kg) at the Army Training Centre, Pirbright for 8-days in week ten of training. Macro and micronutrient content were estimated using dietary analysis software (Nutritics, Dublin) and assessed via an independent sample t-test to establish if there was a sex difference in daily energy, macro or micronutrient intakes.

**Results:**

Estimated daily energy intake was less than the MDRV for both men and women, with men consuming a greater amount of energy compared with women (2846 ± 573 vs. 2207 ± 585 kcal·day^− 1^, *p* < 0.001). Both sexes under consumed carbohydrate (CHO) when data was expressed relative to body mass with men consuming a greater amount than women (4.8 ± 1.3 vs. 3.8 ± 1.4 g·kg^− 1^·day^− 1^, *p* = 0.025, ES = 0.74). Both sexes also failed to meet MDRVs for protein intake with men consuming more than women (1.5 ± 0.3 vs. 1.3 ± 0.3 g·kg^− 1^·day^− 1^, *p* > 0.030, ES = 0.67). There were no differences in dietary fat intake between men and women (1.5 ± 0.2 vs. 1.5 ± 0.5 g·kg^− 1^·day^− 1^, *p* = 0.483, ES = 0.00).

**Conclusions:**

Daily EI in men and women in Phase One training does not meet MDRVs. Interventions to increase macronutrient intakes should be considered along with research investigating the potential benefits for increasing different macronutrient intakes on training adaptations.

## Introduction

British Army standard entry Phase One training is a 14-week training syllabus that includes physical training, field exercises and training on a variety of military-specific skills including load carriage, marching, military drill and weapon and equipment handling [1]. It is characterised by high rates of injury and medical discharge (MD) [1–3]. In Phase One training the overall rate of injury is 0.07 people injured per 100 person-days and that the overall MD rate is 0.02 people injured per 100 person-days [2]. Recruits are exposed to high daily training loads and energy expenditures (EE) which, without adequate nutrient provision, may contribute to a reduction in mood state [4] compromised physical performance, increased musculoskeletal injury (MSKi) risk [5, 6] and medical discharge (MD). Estimated daily EE and training distance covered in Phase One training in men has been reported to be ~ 4000 kcal and 13.5 ± 6.6 km and in women was ~ 3000 kcal and 11.8 ± 4.9 km for men and women, respectively [1]. Women are at greater risk of MSKi during British Army Phase One training and this is supported by evidence that demonstrates women to be 2–3 times at greater risk of injury [2]. The increased risk is not due to sex differences per se but likely due to lower aerobic fitness levels in women, resulting in higher internal load [1, 2, 7]. Therefore, women may require additional dietary support, such as energy and/or protein intake, to facilitate skeletal muscle repair and support the higher training load compared to men [1]. To date, however, there is no suggestion that separate protein intakes should be recommended for men and women. To maintain muscle mass, strength and performance during periods of substantial metabolic demands and concomitant negative energy balance it is recommended a protein intake of at least 1.5 g·kg^− 1^·d^−1^is consumed [8].

In response to a similar training load women have been shown to have greater fatigue resistance and maintenance of muscle function to men [9]. Following a loaded march during British Army training, men had a greater loss in maximal voluntary contractions (MVC) of the knee extensors than women (12 ± 9% vs. 9 ± 13%, *p* = 0.03). The authors suggested that this may have been due to woman possessing a greater proportion of type 1 muscle fibres in the knee extensor muscles. Nevertheless, the MVC and vertical jump height of men following load carriage was still higher than the pre-exercise values for the women and therefore, muscle performance rather than fatigability per se, may contribute to the sex difference in injury incidence [2, 9]. The higher baseline values in the men perhaps allows for greater degradation [9]. Therefore, the lower baseline values in the women may indicate a requirement for nutritional interventions to enhance skeletal muscle recovery. Women may also require other dietary interventions to support training, particularly as recent evidence has shown women to under consume various micronutrients such as iron and calcium, during military training by 77 and 75%, respectively [10].

Dietary intake should match energy expenditure to maintain health and performance and evidence to support this has been extensively reviewed [11–13]. Specifically, an inadequate energy intake (EI) is harmful to performance [4], bone health [5, 14, 15], immune function [16], cognition [17], mood [4] and MSKi risk [5]. It has therefore been recommended to consume 3100–4100 kcal·d^− 1^, specific to Phase one training [18]. Moreover, a negative energy balance of > 500 kcal·d^− 1^ is detrimental to health in the longer term. It has been shown that an energy deficit of this magnitude suppresses the hormone milieu, reduces thyroid function and reduces exercise performance by 9.8% [19]. Reduced thyroid function is of particular concern in military populations due to the suppression on bone formation markers and subsequent risk of a stress fracture [20]. In a crossover study, endurance trained runners undergoing an intense 11-day training programme whilst habitually consuming a diet lower in CHO (5.4 g·kg^− 1^·d^− 1^) experienced a greater deterioration in global mood scores, than when consuming a diet with a higher CHO content (8.5 g·kg^− 1^·d^− 1^) [4]. In military populations it is generally found that soldiers fail to meet recommended energy and nutrient intakes [10, 21–27]. McAdam et al. (2018) found that recruits undergoing Basic Training in the United States (U.S.) experienced a 595 ± 896 kcal·d^− 1^ deficit and 70% of recruits consumed less than the lower limit (6 g·kg^− 1^·d^− 1^) for recommended carbohydrate intake (CHO). Given the large standard deviation for energy intake (896 kcal·d^− 1^) some recruits would have been in a larger energy deficit across the training phase. It is possible that this deficit was underestimated due to the use of an accelerometer to quantify EE. Energy expenditure was estimated via an Actigraph wGT3X monitor using the Sasaki equation which has been shown to have a mean bias of − 0.23 compared to indirect calorimetry [28]. It is also possible that EI was also underestimated due to an acute food diary collection period being used for analysis [29]. In the United Kingdom (UK), the Scientific Advisory Committee on Nutrition (SACN) have developed Military Dietary Reference Values (MDRVs) for British Army recruits [18], but no study has yet quantified dietary intake to establish if these are habitually met.

The aim of this study was therefore to quantify energy, macro and micronutrient intake of British Army recruits to determine if these were adequate compared to MDRVs and Recommended Daily Allowances (RDA). A secondary aim was to compare dietary intake between sexes to establish if future dietary interventions during training needed to be sex specific. Based on other studies in military populations, we hypothesized that men and women would not meet MDRVs for energy intake and that women would be at greater risk of nutrient deficiency compared to men due to a lower energy intake. The findings of this investigation will provide novel data into the nutritional intake of British Army recruits in Phase One training. This data may be used to inform future interventions aimed at improving nutrient intake in this population during British Army training.

## Materials/ methods

### Ethical approval

This study was approved by the U.K. Ministry of Defence Research Ethics Committee (MODREC). For inclusion, recruits at the Army Training Centre Pirbright (ATC(P)), Surrey, UK in week ten of training, were invited to take part. Interested participants received verbal explanation of the study from the research team and provided written informed consent. Twenty-eight women (mean ± SD: age 21.4 ± 3.0 yrs., height: 163.7 ± 5.0 cm, body mass 65.0 ± 6.7 kg, body mass index: 24.2 ± 2.6 kg·m^2^) and seventeen men (mean ± SD: age 20.4 ± 2.3 yrs., height: 178.0 ± 7.9 cm, body mass 74.6 ± 8.1 kg, body mass index: 22.5 ± 1.7 kg·m^2^) volunteered for this study, which was conducted in accordance with the declaration of Helsinki.

### Study design

This was an observational cross-sectional study over an 8-day period. Sample size was based on a priori power analysis using G*power (v3.1.9.2, Dusseldorf) based on previously collected energy intake data in the literature [26]. It was determined that 24 participants (12 men and 12 women) were required to replicate the highest significant effect size of 1.05 for a between-sex difference in energy intake using α = 0.05, β = 0.80. Participant demographics were collected on day one and diet analysis was collected on each day (days 1 to 8).

### Physical characteristics

Height (cm) and body mass (kg) were recorded with recruits wearing Army uniform except for boots using a seca 213 mobile stadiometer and pre-calibrated seca flat scales (Hamburg, Germany).

### Diet logs

Dietary intake was recorded using researcher-led food weighing at breakfast, lunch and dinner in the training centre dining facility. On arrival, participants chose their food and each portion was weighed using pre-calibrated food scales (Salter, 1066 BKDR15, Kent, UK). After each meal, participants were instructed to leave food discards so that these could also be weighed and subtracted from the original weight; to give the actual food portion consumed for that meal [30]. To capture dietary intake between meals and off-camp, participants completed food diaries following guided instructions and estimated the portion size using practical measures (1 cup, 2 handfuls, 1 palm size etc.) [29] and kept any snack or ration discards in discard bags to cross examine against food diaries. Participants were briefed on how to accurately complete a food diary and these were then checked by a member of the research team each day to clarify any unclear information.

### Nutritics analysis

Food records were entered into nutritional analysis software (Nutritics, Dublin, Ireland) for the generation of mean daily energy, macronutrient and micronutrient intakes using the UK Scientific Advisory Committee on Nutrition (SACN) database. The recipes of foods which did not already exist in the database (i.e. ration pack foods) were manually entered using the recipe or nutritional content information provided by the caterer. All data was inputted by the same researcher to reduce data processing variability [31].

### Data presentation and statistical analysis

Physical characteristics and mean nutrient intakes were compared between sexes using an independent samples t-test. Prior to this, dietary intake data was tested for normality using a Shapiro-Wilks test (IBM SPSS v24). Where data showed a significant deviation from a normal distribution, a non-parametric equivalent (Mann Whitney U test) was used. Cohen’s d effects (small = 0.2, medium = 0.5, large = 0.8) were calculated for differences in nutrient intakes between men and women. Following an appropriate Bonferroni adjustment, an alpha level of *p* < 0.001 was set.

## Results

### Physical characteristics

There was a statistically significant difference between sexes in stature (t [22]= 6.521, *p* = < 0.001) and body mass (t [32]= 3.920, *p* = < 0.001) but not age (Z = − 1.126, *p* = .260) or BMI (t [32]= − 1.224, *p* = 0.228).

### Energy intake

There was a statistically significant difference between sexes with men consuming more than women (t [32]=3.508, *p* = 0.001, ES = 1.10). Both men and women consumed less than the MDRVs, with men consuming 69% and women consuming 72% of the recommended energy intake (Table 2). When data was expressed as relative to body there was no differences in energy intake between sexes (t [32]=1.396, *p* = 0.170, ES = 0.46) (Table 2).

### Macronutrient intake

Compared to the MDRVs, men and women under consumed CHO and protein with men consuming a greater absolute total daily amount of CHO than women (Z = -3.708, *p* < 0.001, ES = 1.27). Men also consumed a greater total daily amount of protein than women (Z = -3.708, p < 0.001, ES = 1.28). Total fat intake was not different between sexes t [32]=1.113, *p* = 0.272, ES = 0.37) but under consumed by men. Men consumed a greater amount of fibre than women (t [32]=2.422, *p* = 0.020, ES = 1.16) (Table 1). When data was expressed relative to body mass there was no difference between sexes for CHO (t [32]=2.333, *p* = 0.025, ES = 0.74), protein (t [32]=2.241, *p* = 0.030, ES = 0.67), fat (t [32]= − 0.708, *p* = 0.483, ES = 0.00) or fibre intake (t [32]=0.840, *p* = 0.406, ES = 0.00) (Table 2).

**Table 1:** Absolute nutrient intake for participants compared to MDRVs and RDA

Nutrient	All	Men	MDRV	Women	MDRV
Energy (kcal·day^− 1^)	2439 ± 653	2846 ± 573*	4100.0	2207 ± 585*	3100.0
CHO (g·day^− 1^)	283 ± 98	352 ± 92*	513–615	243 ± 79*	388–465
PRO (g·day^− 1^)	94 ± 27	114 ± 29	123–154	83 ± 18	93–116
Fat (g·day^− 1^)	103 ± 25	109 ± 21	128–159	100 ± 27	96–121
Fibre (g·day^− 1^)	20 ± 6	23 ± 5	30	18 ± 1	30
Calcium (mg·d^− 1^)	837.0 ± 383.0	1078.0 ± 418.0*	700.0	699.0 ± 287.0*	700.0
Copper (mg·d^− 1^)	0.9 ± 0.3	1.0 ± 0.0	1.2	0.9 ± 0.3	1.2
Folate (μg·d^− 1^)	173.0 ± 84.0	231.0 ± 95.0*	200.0	140.0 ± 55.0*	200.0
Iodine (μ·d^− 1^)	99.0 ± 64.0	135.0 ± 79.0	140.0	77.0 ± 44.0	140.0
Iron (mg·d^− 1^)	8.7 ± 3.0	10.0 ± 3.0*	8.7	7.0 ± 2.0*	14.8
Magnesium (mg·d^− 1^)	198.0 ± 77.0	239.0 ± 94.0	300.0	174.0 ± 55.0	270.0
Niacin (mg·d^− 1^)	14.8 ± 6.3	19.9 ± 7.3*	16.5	12.0 ± 3.0*	13.2
Phosphorus (mg·d^− 1^)	997.0 ± 382.0	1227.0 ± 461.0	550.0	865.0 ± 254.0	550.0
Potassium (mg·d^− 1^)	2386.0 ± 877.0	2859.0 ± 1051.0	3500.0	2115.0 ± 634.0	3500.0
Riboflavin (mg·d^− 1^)	1.1 ± 0.7	1.6 ± 0.8*	1.3	0.8 ± 0.4*	1.1
Selenium (μg·d^− 1^)	39.0 ± 21.0	57.0 ± 25.0	75.0	29.0 ± 11.0	60.0
Sodium (g·d^− 1^)	2.7 ± 0.7	3.0. ± 0.6	2.4	2.5 ± 0.7	2.4
Thiamin (mg·d^− 1^)	1.3 ± 0.5	1.5 ± 0.5	1.0	1.1 ± 0.3	0.8
Vitamin A (μg·d^− 1^)	634.0 ± 410.0	840.0 ± 388.0	700.0	516.0 ± 380.0	600.0
Vitamin B_12_ (μg·d^− 1^)	4.3 ± 2.6	6.0 ± 3.2*	1.5	3.3 ± 1.3*	1.5
Vitamin B_6_ (mg·d^− 1^)	1.5 ± 0.9	2.0 ± 1.2	1.4	1.2 ± 0.3	1.2
Vitamin C (mg·d^− 1^)	55.0 ± 38.0	67.0 ± 49.0	40.0	49.0 ± 29.0	40.0
Vitamin D (μg·d^− 1^)	2.0 ± 1.0	2.0 ± 1.0	10.0	1.0 ± 1.0	10.0
Zinc (mg·d^− 1^)	7.1 ± 2.5	8.0 ± 3.0	9.5	6.0 ± 1.0	7.0

Energy, macronutrient and micronutrient intake of all participants and with data grouped according sex to establish differences in intakes. Intakes for each sex were then compared to recommendations. The recommended MDRV for CHO, protein and fat towards total energy intake is 50-60%, 12-15% and 28-35%, respectively. These values were used to calculate the absolute amount of CHO, protein and fat needed to achieve the required energy intake in men (4100 kcal·d^-1^) and women (3100 kcal·day^-1^). *EI* Energy intake , *MDRV* Military Dietary Reference Value , *CHO* Carbohydrate , grams·body mass^-1^·day^-1^ (g·kg^-1^·d^-1^), milligrams·day^-1^ (mg·d^-1^) and micrograms·day^-1^ (μg·d^-1^). *indicates a statistically significant differecnce between sexes

**Table 2:** Relative daily nutrient intakes for participants compared to MDRVs/RDA and Sport Nutrition guidelines

Nutrient	All	Men	MDRV	Women	MDRV
Energy (kcal·kg^− 1^·d^− 1^)	35.7 ± 9.5	38.3 ± 7.3	55.0	34.2 ± 10.3	48.0
CHO (kcal·kg^− 1^·d^− 1^)	4.1 ± 1.4	4.8 ± 1.3	6.0–8.0	3.8 ± 1.4	6.0–7.0
PRO (kcal·kg^− 1^·d^− 1^)	1.4 ± 0.4	1.5 ± 0.3	1.6–2.0	1.3 ± 0.3	1.4–1.8
Fat (kcal·kg^− 1^·d^− 1^)	1.5 ± 0.4	1.5 ± 0.2	1.7–2.1	1.5 ± 0.5	1.5–1.9
Fibre (kcal·kg^− 1^·d^− 1^)	0.3 ± 0.1	0.3 ± 0.1	0.4	0.3 ± 0.1	0.5
Calcium (mg·kg^− 1^·d^− 1^)	12.17 ± 5.18	14.43 ± 5.21	9.38	10.88 ± 4.78	10.80
Copper (mg· kg^− 1^·d^− 1^)	0.01 ± 0.01	0.01 ± 0.01	0.02	0.02 ± 0.01	0.02
Folate (μg· kg^− 1^·d^− 1^)	2.50 ± 1.12	3.08 ± 1.19	2.68	2.18 ± 0.95	3.08
Iodine (μg· kg^− 1^·d^− 1^)	1.44 ± 0.91	1.83 ± 1.06	1.88	1.22 ± 0.74	2.15
Iron (mg· kg^− 1^·d^− 1^)	0.12 ± 0.04	0.15 ± 0.04	0.12	0.12 ± 0.04	0.23
Magnesium (mg·kg^− 1^·d^− 1^)	2.90 ± 1.07	3.22 ± 1.21	4.02	2.72 ± 0.96	4.15
Niacin (mg· kg^− 1^·d^− 1^)	0.41 ± 0.15	0.51 ± 0.17	0.22	0.36 ± 0.09	0.20
Phosphorus (mg·kg^− 1^·d^− 1^)	14.54 ± 5.18	16.46 ± 5.89	7.37	13.45 ± 4.48	8.46
Potassium (mg· kg^− 1^·d^− 1^)	34.74 ± 11.86	38.42 ± 13.70	46.91	32.63 ± 10.35	53.85
Riboflavin (mg· kg^− 1^·d^− 1^)	0.02 ± 0.01	0.02 ± 0.01	0.02	0.01 ± 0.01	0.02
Selenium (μg· kg^− 1^·d^− 1^)	0.57 ± 0.29	0.77 ± 0.35	1.00	0.46 ± 0.18	0.92
Sodium (g· kg^− 1^·d^− 1^)	0.04 ± 0.01	0.04 ± 0.01	0.03	0.04 ± 0.01	0.04
Thiamin (mg· kg^− 1^·d^− 1^)	0.02 ± 0.01	0.02 ± 0.01	0.01	0.02 ± 0.01	0.01
Vitamin A (μg· kg^− 1^·d^− 1^)	9.25 ± 5.94	11.34 ± 5.12	9.38	8.05 ± 6.13	9.23
Vitamin B_12_ (μg·kg^− 1^·d^− 1^)	0.06 ± 0.04	0.08 ± 0.04	0.02	0.05 ± 0.02	0.02
Vitamin B_6_ (mg· kg^− 1^·d^− 1^)	0.02 ± 0.01	0.03 ± 0.02	0.02	0.02 ± 0.01	0.02
Vitamin C (mg·kg^− 1^·d^− 1^)	0.81 ± 0.54	0.89 ± 0.62	0.54	0.77 ± 0.49	0.62
Vitamin D (μg· kg^− 1^·d^− 1^)	0.03 ± 0.02	0.03 ± 0.02	0.13	0.03 ± 0.02	0.15
Zinc (mg· kg^−1^·d^− 1^)	0.10 ± 0.03	0.12 ± 0.04	0.13	0.10 ± 0.03	0.11

Daily nutrient intakes of all participants with data separated for sex to establish relative differences. Data was compared to recommendations. Military dietary reference values (MDRVs) were used for energy, macro and micronutrient intake recommendations. Recommended relative intakes were calculated by dividing the recommended absolute intake by the average body mass for each sex. Data presented as mean ± standard deviations. No statistically significant differences between sexes were observed.

### Micronutrient intake

When compared to men, women consumed less calcium (t [32]= 3.645, *p* = 0.001, ES = 1.06),iron (t [32]=4.262, *p* < 0.001, 1.18), sodium (t [32]=2.700, *p* = 0.010, ES = 0.77), vitamin B_6_ (Z = -3.123,*p* = 0.002, ES = 0.91), vitamin B_12_ (Z = -3.477,*p* = 0.001, ES = 1.11), potassium (Z = -2.537, *p* = 0.011, ES = 0.86), niacin (Z = -4.062, *p* < 0.001,ES = 1.42), iodine (Z = -2.733, *p* = 0.006, ES = 0.91), thiamine (Z = -2.355, *p* = 0.010), riboflavin (Z = -3.576, *p* < 0.001, ES = 0.97), phosphorus (Z = -2.976, *p* = 0.003, ES = 0.97) and folate (Z = -3.391, *p* = 0.001, ES = 1.17). Men and women consumed less than the RDA for copper, magnesium and vitamin D with women consuming significantly less magnesium (Z = -2.464, *p* = 0.014, ES = 0.84) and vitamin D (Z = -2.257, *p* = 0.024, ES = 1.00) but not copper (t [32]=1.035, *p* = 0.306, ES = 0.47). Women consumed an inadequate amount of vitamin A when compared to the RDA and this was significantly less than men (Z = -2.562, *p* = 0.010, ES = 0.84). Both men and women consumed adequate amounts of vitamin C when compared to the RDA with no differences between sexes (Z = -1.049, *p* = 0.294, ES = 0.45). When micronutrient data was expressed relative to body mass there was no difference for iron (t [32]=2.468, *p* = 0.18, ES = 0.75), calcium (t [32]=2.28, *p* = 0.027, ES = 0.71), magnesium (t [32]=1.513, *p* = 0.138, ES = 0.46), vitamin A (t [32]=1.808, *p* = 0.078, ES = 0.58), vitamin C (t [32]=0.289, *p* = 483, ES = 0.21), vitamin B_12_ (t [42] = 3.043, *p* = 0.004, ES = 0.95), phosphorus (t [32]=1.913, *p* = 0.063, ES = 0.58), potassium (t [32]=1.584, *p* = 0.121, ES = 0.48), selenium (t [19.791] = 3.351, *p* = 0.003, ES = 1.11), sodium (t [32]=0.733, *p* = 0.468, ES = 0.00), zinc (t [32]=0.2130, *p* = 0.039, ES = 0.57), iodine (t [32]=2.228, *p* = 0.031, ES = 0.67), niacin (t [20.989] = 3.249, *p* = 0.004, ES = 1.10), folate (t [32]=2.756, *p* = 0.009, ES = 0.70), vitamin D (Z = − 1.786, 0.074, ES = 0.00), vitamin B_6_ (Z = -1.837, *p* = 0.066, ES = 0.63), copper (Z = -0.266, *p* = 0.790, ES = -0.45), thiamine (Z = -1.102, *p* = 0.271, ES = 0.00) or riboflavin (Z = − 2.807, *p* = 0.005, ES = 0.57) (Table 2).

## Discussion

The aim of this study was to quantify daily energy, macro and micronutrient intake of British Army recruits in Phase One training and to compare intakes between men and women. Our primary finding was that men and women under consumed daily energy intake by ~ 1200 and ~ 800 kcal·d^− 1^, respectively when compared to MDRVs. The MDRVs are based on measurements of daily energy expenditures via the doubly labelled water method in a similar cohort within this population whilst undertaking the same programme in British Army Phase One training [18]. The reported underconsumption of daily energy intake in this population observed in this study is typical of military populations and values estimated here are similar to other research [21, 23, 25–27]. The observed under consumption of total calories in this study, meant that recruits did not meet MDRV and RDAs for specific macro and micronutrients. Furthermore, due to a lower daily energy intake of women compared to men, and higher RDA for some micronutrients (i.e. iron), women are at greater risk of inadequate intakes when compared to guidelines and need to increase habitual iron intake by ~ 53% to meet the RDA of 14.8 mg·d^− 1^ (Table 1).

Energy intake of men and women in this study was inadequate when compared to MDRVs (Table 1) and this may increase the incidence of reduced energy availability [33] which, in turn, may in increase the risk of injury [5, 14]. Reduced chronic energy availability may lead to impaired physiological functions such as metabolic rate, protein synthesis, bone health, menstrual function and cardiovascular health [33]. Musculoskeletal injury risk (MSKi) may be increased during periods of reduced energy availability with concomitant reductions in skeletal muscle mass are observed due to reduced protein turnover [34, 35] Furthermore, skeletal muscle response to the training stimulus maybe downregulated during periods of reduced energy availability. For example, a daily energy surplus of ~ 358–478 kcal·d^− 1^ is recommended to maximise muscle hypertrophy with resistance training [36]. Energy restriction has been shown to downregulate mTOR signalling activity and this is likely due to the inhibited protein translation and subsequently lower phosphorylation of protein kinase B (AKt), the mammalian target of rapamycin (mTOR), ribosomal protein S6 kinase (P70S6K) and ribosomal protein S6 (rps6) [37]. An energy deficit of ~ 40% upregulates mRNA of the skeletal muscle ubiquitin proteasome system (UPS) which regulates skeletal muscle proteolysis [38]. Our data demonstrates men and women consumed adequate energy to prevent an estimated deficit vs. the MDRVs of ≤40% and consumed ~ 31% and ~ 29% less than the MDRVs, respectively, which may still be considered as a considerable energy deficit. In relation to bone health, reduced energy availability reduces calcium absorption, bone turnover and bone mineral density [5], and thus, increases stress fracture risk [39] with women appearing to be more affected than men [40]. Furthermore, a reduced energy availability will increase the risk of inadequate supply of macro and micronutrients, which will likely impair physical performance and increase the risk of injury further [6].

Men and women both consumed less than the minimum recommended intake for CHO compared to MDRVs (Table 1-2). These results are similar to intakes of U.S. Army personnel, which found ~ 70% of personnel consumed less than 6 g·kg^− 1^·d^− 1^ carbohydrate [27]. Given that participants undergoing Phase One training have energy expenditures between ~ 3000 to ~ 4000 kcal·d^− 1^ [1] which is comparable to athletes in team sports [41] it may be appropriate to aim for similar CHO intakes per day (5–7 g·kg^− 1^·d^− 1^) [42]. As such, British Army recruits may not be maintaining muscle glycogen stores to support training. Lower intakes of CHO during intensified training periods have been shown to reduce exercise performance and mood state in athletes [4] and contribute to immunosuppression [32]. Sub-optimal intakes of CHO during hard training periods in athletes, increases concentrations of cortisol whilst attenuating the secretion of immunoglobin-A (SlgA), and thus, increases the risk of contracting an upper respiratory tract infection [32, 43]. Taken together, CHO intakes below recommended intakes whilst undergoing military training may result in missed training days and possibly failure to complete training due to increased illness and injury risk. Future research should assess the effects of additional CHO intake on training outcomes, illness and injury incidence. Furthermore, research investigating the impact of nutrient timing in this population is also warranted given the influence this may have on recovery, tissue repair, muscle protein synthesis and psychological mood [44]. It has been shown that British Army officer cadets may under consume suboptimal levels of CHO and protein between mealtimes [45] but data in the recruit population is currently lacking.

Protein intakes in men and women were less than the MDRVs but were in-line with sport nutrition guidelines (1.2–2.0 g·kg^− 1^·d^− 1^) [12] although women did have a lower relative intake than men (Table 2). To date, however, specific protein intakes are not recommended for British Army recruits. Intakes in the range of 1.2–2.0 g·kg^− 1^·d^− 1^ are recommended in athletes to support metabolic adaptation, repair, remodelling, and for protein turnover [12]. Despite both sexes meeting this range in this study, it should be noted that intakes were at the lower end of this, and that true protein requirements may be at the upper limit of this range to meet training demands (1.5–2.0 g·kg^− 1^·d^− 1^). In fact, evidence now suggests endurance athletes require more than the original recommended intake of 1.2–1.4 g·kg^− 1^·d^− 1^ and instead should consume 1.6–1.8 g·kg^− 1^·d^− 1^ on intense training days [46]. Given the arduous nature of military training and that military type exercise (i.e. load carriage) stimulates muscle protein synthesis more than endurance type exercise (i.e. running) [47], military personnel may require a daily protein intake of ≥1.5 g·kg^− 1^ [8]. Furthermore, intakes of > 2.0 g·kg^− 1^ during energy restriction may be needed to maximise the loss of fat-mass whilst also maintaining lean-tissue mass [13]. A protein intake higher than that observed in the current study has been shown to have physiological and performance benefits [48–50]. A protein intake of 3.0 g·kg·^− 1^d^− 1^ resulted in a 30% possibility that the decrement in time trial performance pre-and-post the intervention was attenuated vs. a moderate protein intake (1.5 g·kg^− 1^·d^− 1^) [49]. U.S. Marines who were supplemented daily with protein (12 g protein, 9.6 g CHO, 3.6 g fat) for 54-days had 14% fewer visits to the medical centre compared to the placebo group (0 g protein, 9.6 g CHO, 3.6 g fat) and 40% less visits to the medical centre compared to the control group [48]. More recently, U.S. Army soldiers participating in Initial Entry Training who supplemented daily with whey protein (77 g, 580 kcal) had a greater reduction of fat-mass (− 4.5 kg, Cohen’s d = − 0.67 vs. -2.7 kg, Cohen’s d = − 0.40) compared to a group who supplemented daily with CHO (127 g, 580 kcal). The total daily protein intake was 2.8 g·kg^− 1^·d^− 1^ in the protein group, which is far greater than both men and women in the current study (Table 2) [50]. An increased protein intake > 1.5 g·kg^− 1^·d^− 1^ may also have psychological benefits. Endurance trained cyclists undergoing three weeks of high-intensity training had a 97% chance that a higher protein intake (3 g·kg^− 1^·d^− 1^) attenuated increased symptoms of stress compared to a moderate protein intake (1.5 g·kg^− 1^·d^− 1^) when participants were weight stable and when CHO intake was matched between conditions (6 g·kg^− 1^·d^− 1^) [49]. This provides the rationale that protein intake should be considered in relation to other functions other than muscle protein synthesis and that a daily protein intake > 1.5 g·kg^− 1^ may provide psychological benefits to individuals undergoing intense training (i.e. military training). Given the apparent benefits on increasing dietary protein to > 2.0 g·kg^− 1^·d^− 1^ in periods of arduous training, it should be investigated if an additional protein intake to that of habitual intakes in British Army recruits in Phase One training influences training adaptions and training outcomes.

The total micronutrient intake data for the cohort showed that there was an inadequate intake of magnesium, vitamin D, potassium, selenium, copper, iodine and folate (Table 1-2). Similarly, data collected in men and women during Basic Combat Training in the U.S. Army showed an inadequate intake of vitamin D, magnesium and potassium with women under consuming calcium and iron [10]. Given the reported intake of calcium (699 ± 287 mg·d^− 1^) and iron (7 ± 2 mg·d^− 1^) in women in this study, the risk of an inadequate intake of these micronutrients in this population is highlighted. Previously, it has been observed that training increase bone mineral content (BMC) and bone mineral density (BMD) of the arms, legs and pelvis in men and women undergoing the same training course at ATC(P). Conversely, it was observed training reduced BMC for the trunk and ribs and BMD for the ribs in both men and women (unpublished observations). These changes in BMD and BMC may be explained by habitual calcium intakes (837 ± 383 mg·d^− 1^) with some consuming less than the RDA as shown by the reported standard deviation. Furthermore, it has previously been reported that only 9% of men and 36% of women entering Phase one training are vitamin D sufficient [51]. Given the inadequate intake of vitamin D and calcium, it should be investigated if increasing the intake of these micronutrients benefits training outcomes. For instance, female U.S. Navy recruits undergoing basic training who supplemented daily with 2000 mg calcium and 800 IU vitamin D had a 21% reduction in stress fracture incidence compared to a control group [52]. It is unknown, however, if the reduction was due to an increased calcium or vitamin D intake. The low habitual intake of iron in women compared to the RDA (Table 1-2) is comparable to that of their U.S. Army counterparts [10]. British Army training appears to have a deleterious effect on iron status with ferritin and haemoglobin decreasing significantly pre and post Phase One training in men and women. Ferritin has been shown to reduce from 105.1 to 78.7 μg·L^− 1^ in men and from 52.7 to 47.7 μg·L^− 1^ in women. Haemoglobin has been shown to reduce from 149.7 to 147.1 g·dL^− 1^ in men and from 139.2 to 132.1 g·dL^− 1^ in women in 14 weeks of training. These changes in iron status contributed to a 6.9 and 2.3% development of anaemia in women and men, respectively [53]. As such, research investigating iron requirements and the potential benefits of iron supplementation in British Army recruits undergoing Phase One training may be warranted. It is possible that recruits may require 70% more than the RDA [12]. For example, similar to athletes, British Army recruits who engage in regular exercise increase hepcidin levels which then inhibits iron absorption and contributes to a decrease in iron status [54]. Therefore an intervention may be to increase dietary iron intake particularly during periods not in close proximity to exercise to promote iron absorption and thus iron status [12].

## Conclusion

Energy intake in men and women in British Army Phase One training is inadequate when compared to MDRVs. When considered to MDRVs, men and women both under consume CHO and protein and therefore interventions to combat this should be considered. Given this and the potential benefits of increasing protein intakes above 1.5 g·kg^− 1^·d^− 1^ in military populations, future research investigating this should be explored. Furthermore, research aiming to better understand habitual protein requirements may be warranted. Given the low vitamin D intakes in both sexes and low iron and calcium intakes in women, research investigating the effects of micronutrient supplementation on training outcomes is needed. Finally, research which investigates changes in habitual dietary intake during Phase One training should be considered as well as data on the timing of daily energy and macronutrients intakes due to the potential effects on training adaptations and the implications of nutritional based interventions.

## Data Availability

Datasets used and/or analysed during the study are available from the corresponding author in reasonable request.

## Abbreviations

Akt: Protein kinase B
ATC(P): Army training centre pirbright
BMC: Bone mineral content
BMD: Bone mineral density
CHO: Carbohydrate
EE: Energy expenditure
EI: Energy intake
Kcal: Kilocalorie
LBM: Lean body mass
MD: Medical discharge
MDRV: Military dietary reference values
MODREC: Ministry of defence research ethics committee
MRNA: Messenger RNA
MSKi: Musculoskeletal injury risk
mTOR: Mammalian target of rapamycin
P70S6K: Ribosomal protein S6 kinase
RDA: Recommended daily allowance
rps6: Ribosomal protein S6
SACN: Scientific advisory committee on nutrition
SD: Standard deviation
SIgA: Secretory immunoglobulin A
U.K.: United Kingdom
U.S.: United States
